# Substrate types and applications of MXene for surface-enhanced Raman spectroscopy

**DOI:** 10.3389/fchem.2024.1378985

**Published:** 2024-03-12

**Authors:** Zhi-Wei Liu, Gong Wang, Yun-Fei Li, Yu Yu

**Affiliations:** ^1^ Center for Advanced Laser Technology, Hebei University of Technology, Tianjin, China; ^2^ Innovation and Research Institute of Hebei University of Technology in Shijiazhuang, Shijiazhuang, China; ^3^ Hebei Key Laboratory of Advanced Laser Technology and Equipment, Tianjin, China

**Keywords:** surface-enhanced Raman spectroscopy, MXene, biomedical sensing, food safety inspection, environmental detection

## Abstract

Surface-enhanced Raman spectroscopy (SERS) has been widely used in the analysis of analytes because of its unique fingerprint characteristics, high sensitivity, and fast detection response. MXene is widely used in SERS studies among the various substrates due to its ultra-high chemical stability, excellent conductivity, hydrophilicity, and low fabrication cost. This mini-review summarizes MXene’s research in the SERS field from two aspects. We reviewed MXene materials used as SERS substrates alone and combined with noble metal particles primarily. Subsequently, we outlined representative applications of MXene-based SERS in biomedicine, food safety, and environmental monitoring. Moreover, we discussed the technical bottleneck and the prospect of future development in this field.

## 1 Introduction

SERS is a sensitive, fast, and non-destructive molecular vibration spectroscopy technique that has the advantages of sensitive and rapid identification of biomolecules, no sample pretreatment, and low reagent consumption and has become an ideal method for real-time detection of various samples (Qian and Nie, 2008; Shahzad et al., 2016; Xia, 2018). The applications of SERS have been widely realized in several fields, especially in chemical biological analysis (Hu et al., 2014), biological imaging (Carrillo-Carrión et al., 2019), and catalytic process monitoring (Zhang et al., 2017). Of the various factors affecting the effectiveness of SERS in practical applications, the most significant is the substrate. The design and fabrication of high-performance SERS substrates are potent drivers for generating significant Raman signals. Conventional SERS substrates use noble metal nanomaterials, led by Au and Ag, and utilize the powerful localized surface plasmon resonance effect of noble metals to achieve sensitive detection of SERS (Phan-Quang et al., 2019; Wang and Guo, 2020; Xie et al., 2023). Non-noble nanomaterials, as supplements to the noble metal substrates, inject new vitality into the traditional SERS and provide an optimized solution to solve the challenges of the metal substrates in practical applications. Non-noble nanomaterials include MXene (Shahzad et al., 2016; Lin et al., 2017), metal-organic frameworks (MOFs) (Masoomi et al., 2019), graphene and its derivatives (Huang et al., 2015; Cai et al., 2018), transition metal sulfide (Li et al., 2017), and black phosphorus (Pumera, 2017). These materials' distinctive qualities have drawn attention to them, features including high optical qualities, ease of manufacturing, a large specific surface area, and strong biocompatibility (Su et al., 2019). Because of its exceptional mechanical stability, high hydrophilicity, and metal-like electrical conductivity, MXene is one of the non-noble nanomaterials that should raise the most concern (Anasori et al., 2015).

MXenes are commonly represented by the formula M_n+1_X_n_T_X_, in which n ranges from 1 to 3, and T_X_ represents a surface functionality group, such as -OH, =O, -F, and rarely -Cl. Generally, MXenes are synthesized by selective extracting of “A" layers from their precursors MAX phase, where M stands for a transition metal, A represents A III A and IV A group elements, and X is carbon or nitrogen (Naguib et al., 2014). The MXenes family is extensive, including Ti_3_C_2_, Ti_2_C, Nb_2_C, V_2_C, (Ti_0.5_, Nb_0.5_)_2_C, (V_0.5_, Cr_0.5_)_3_C_2_, Ti_3_CN and Ta_4_C_3_ (Naguib et al., 2012; Naguib et al., 2013). They produce distinctive electronic and optical properties by fusing the hydrophilic nature of their termination surfaces with the high metallic conductivity of transition metal carbides and nitrides (Huang et al., 2018). There are many advantages to using MXenes as SERS substrates. First, the ultra-high chemical stability makes them suitable for a variety of chemical analyses and monitoring. Consequently, a variety of MXene-based SERS systems have been shown to detect organic contaminants, including pesticides and dyes. A combination of Ti_3_C_2_T_X_ with gold, silver, and platinum nanoparticles was suggested in one of the early investigations as a sensitive way to detect methylene blue in aqueous solutions using SERS with an enhancement factor of 10^5^ (Satheeshkumar et al., 2016); second, the enhancement mechanism of MXenes through charge transfer (CT) ensures that they achieve high selectivity even in complex samples (Limbu et al., 2020); third, due to the large surface area, excellent electrical conductivity, and hydrophilicity, MXenes can be used to create a range of SERS-based biosensors, these properties help MXene to be combined with other materials, such as precious metals, graphene, and carbon nanotubes, to form more stable materials (Sinha et al., 2018). For example, paper-based SERS substrates were heavily used in water treatment processes due to their large surface area and high potential to accumulate analytes (Soundiraraju and George, 2017a); and lastly, the fabrication of MXene substrates is inexpensive. Therefore, developing high-quality SERS substrates based on MXene can favorably broaden the application potential of SERS for biomedical sensing, food safety detection, environmental monitoring, electrochemical analysis, and other fields with up-and-coming application prospects.

This review offers an in-depth analysis of the development and design of MXene SERS substrates, focusing on recent advances in typical applications. We first describe the two types of using MXene as a SERS substrate alone and in combination with noble metal particles and summarize their preparation strategies. Then, we summarize the most representative application scenarios of MXene in SERS: biomedical sensing, food safety detection, and environmental monitoring. Finally, we discuss the challenges and perspectives of MXene-based SERS substrates regarding the design and refinement of high-quality substrates, environmental friendliness, and the expansion of applications.

## 2 Substrates for surface-enhanced Raman spectroscopy

### 2.1 MXene directly as a substrate material for SERS

**FIGURE 1:**
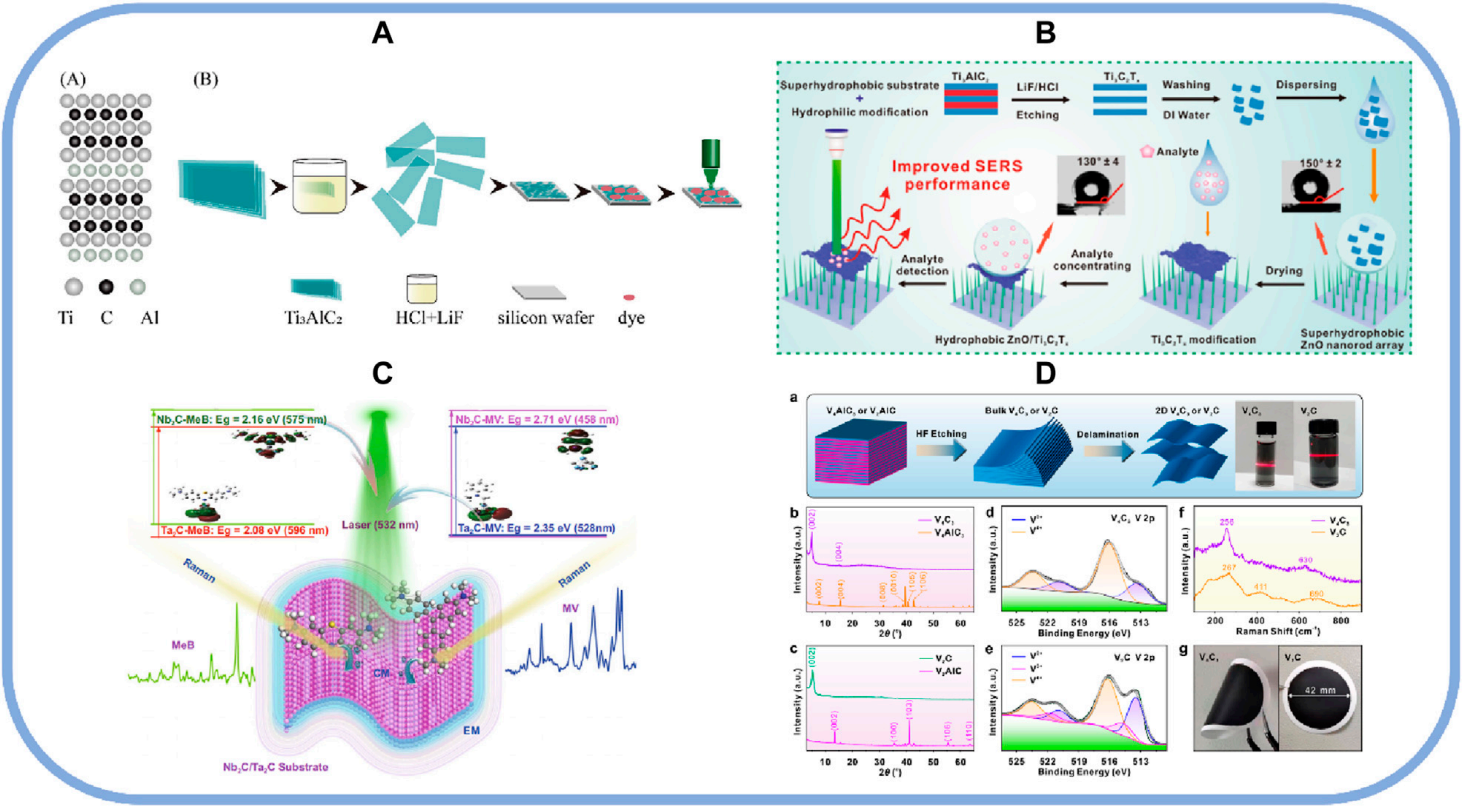
**(A)** Illustration of the fabrication process for Ti_3_C_2_Tx nanosheets and its SERS substrates preparation; reproduced with permission from (Liu et al., 2020b). **(B)** Illustration of the fabrication of a ZnO-Ti_3_C_2_T_X_ substrate; reproduced with permission from (Wu et al., 2023). **(C)** Mechanism diagram of the experimental design for optimizing SERS sensitivity of Nb_2_C and Ta_2_C MXenes; reproduced with permission from (Peng et al., 2021). **(D)** Synthesis and application of V_4_C_3_ and V_2_C to SERS; reproduced with permission from (Lan et al., 2022).

MXenes have recently attracted considerable interest and research because of their excellent metallic conductivity, hydrophilicity, and flexibility (Luo et al., 2022). Unlike noble metal materials, the non-noble material MXene has more abundant physicochemical properties and shows encouraging selectivity and sensitivity, offering great possibilities for optimizing performance (Tyagi et al., 2023). MXene can enhance the Raman signal of organic dyes in solutions and substrates and be directly used as a SERS substrate. In 2017, Ti_3_C_2_T_X_ MXene was first demonstrated to have good SERS activity as a SERS substrate, showing a considerable Raman signal enhancement of R6G (Sarycheva et al., 2017). As a result of charge transfer interactions between the MXene surface and the probe molecule, r-Ti_3_C_2_T_X_ had higher SERS activity than conventional Ti_3_C_2_T_X_. This was achieved by increasing the density of electronic states in the Fermi energy level of r-Ti_3_C_2_Tx enhanced. An alternative method of reducing Ti_3_C_2_T_X_ with l-ascorbic acid at room temperature to obtain r-Ti_3_C_2_T_X_ revealed this (Limbu et al., 2020). The experimental results proved that the application of r-Ti_3_C_2_T_X_ is more promising. As shown in Figure 1A, an optimized preparation method of reducing the ultrasonication duration while adjusting the HCl/LiF ratio generated large-sized and monolayered Ti_3_C_2_T_X_ nanosheets to detect dye molecules at a low detection limit, demonstrating the substrate selectivity between dye molecules and excitation wavelengths. It is worth noting that when stored Ti_3_C_2_T_X_ nanosheets for 1 month, there was no significant change in the SERS signal compared with the newly prepared Ti_3_C_2_T_X_, which confirmed that the large-size Ti_3_C_2_T_X_ substrate still had good uniformity and stability after 1 month (Liu R. et al., 2020). This work lays the foundation for using large-sized Ti_3_C_2_T_X_ as a SERS sensor for the ultrasensitive detection of biomolecules.

Another significant potential advantage of MXene is its ability to deposit materials on different substrates, including flexible materials and metal oxides. When Ti_2_NT_X_ was loaded onto paper, silicon, and glass to prepare different SERS substrates based on them, the results showed that Ti_2_NT_X_ had the highest reinforcement strength on paper-based substrates, up to 10^12^. When compared to flat glass and silicon substrates, the paper substrate’s pleated surface and fiber shape increased SERS activity because of the sample’s inherent roughness (Soundiraraju and George, 2017a). As shown in Figure 1B, the hydrophilic Ti_3_C_2_T_X_ film could be deposited on the superhydrophobic ZnO nanorod array to form SERS substrates with analyte affinity. Thanks to the interfacial charge transfer property, this sample’s limit of detection (LOD) reached 10^−11^ M (Wu et al., 2023), demonstrating the potential of MXene for detecting biomolecules. The SERS activity of MXenes and MXene-based hybrid nanomaterials on substrates has been investigated.

Other members of the MXene family also possess good SERS activity and can be used directly as substrates. As shown in Figure 1C, Nb_2_C and Ta_2_C MXenes were first demonstrated to exhibit significant SERS performance in 2021 with enhancement factors of 3.0 × 10^6^ and 1.4 × 10^6^, respectively, which were synergistically achieved by charge transfer resonance enhancement and electromagnetic enhancement (Peng et al., 2021). SERS detection of multilayered solid-solution TiVC nanosheets synthesized using a one-step chemical etching method showed a stunning femtomolar detection limit for R6G and a SERS intensity of 3.27 × 10^12^. The abundant density of states near the TiVC Fermi energy level and the strong interactions between TiVC and the analytes were responsible for promoting intermolecular charge transfer resonances in the TiVC complexes, resulting in significant Raman enhancements (He et al., 2022). However, researchers tended to focus on modulating the physical and chemical properties of MXene to enhance SERS strength while neglecting to utilize molecular enrichment to improve performance. As shown in Figures 1A, D MXene-based SERS membrane designed using an integrated 2D miniaturization and molecular enrichment strategy enabled fast molecular enrichment, a high molecular removal rate, and ultra-high sensitivity (5 × 10^9^ M) detection. This research investigated two vanadium carbide (V_4_C_3_ and V_2_C) MXenes for ultrasensitive SERS sensing and used vacuum-assisted filtration to enhance the performance further (Lan et al., 2022). Meanwhile, this study also enables an innovative platform for applying MXene materials for non-plasma SERS detection. MXene, as an emerging two-dimensional non-noble material, has been proven to have good SERS activity, and its direct use as a SERS substrate has a broad application prospect in high-sensitivity molecular detection. However, less research has been done on MXenes' stability when used as SERS substrates, which could impede the advancement of MXenes' performance and the use of SERS.

### 2.2 MXene hybrids with noble metal nanoparticles

**FIGURE 2:**
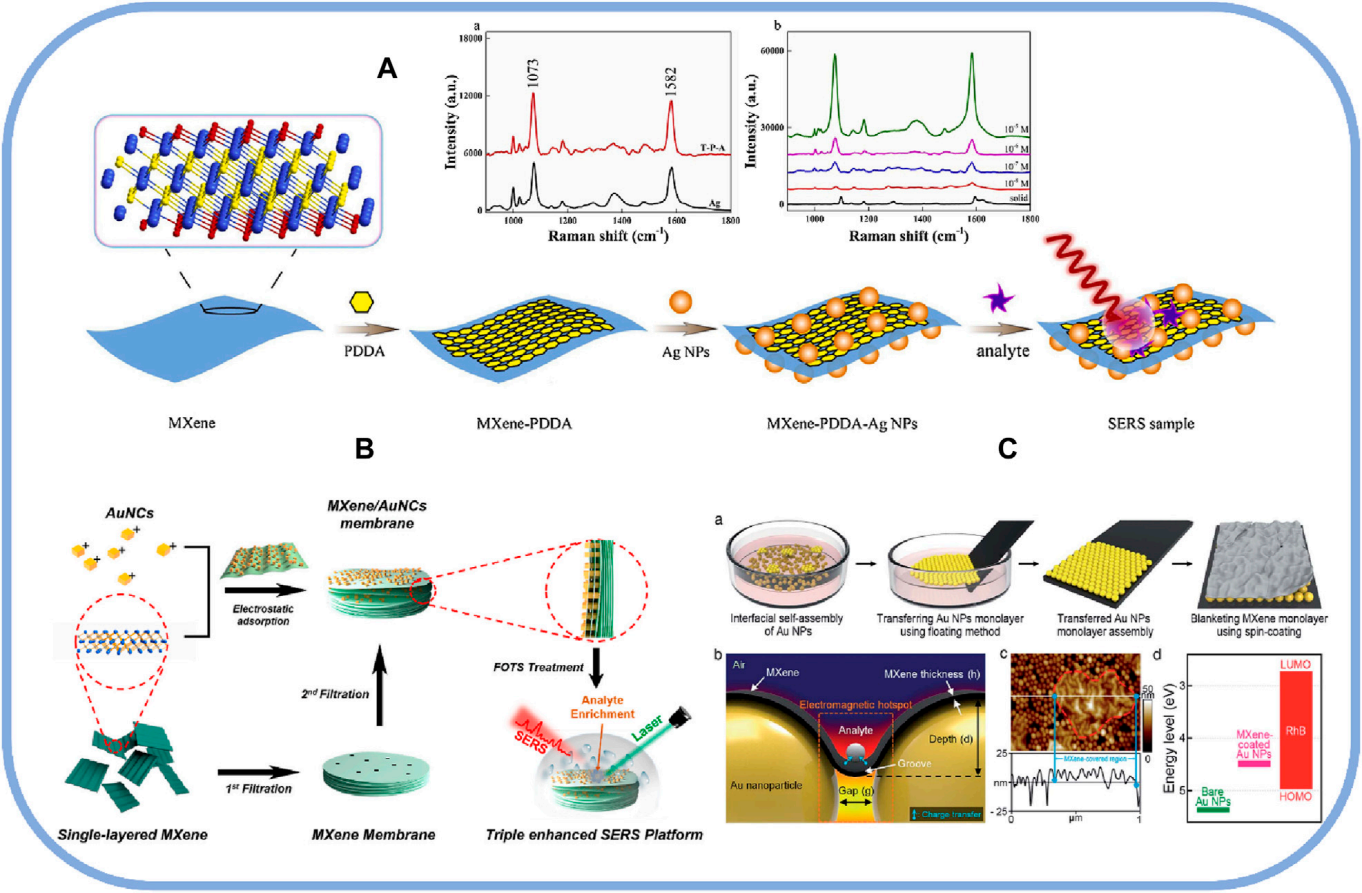
**(A)** Schematic of the preparation and SERS detection of MXene-PDDA-Ag NPs substrates and SERS spectra of different substrates and different 4-MBA molecular concentrations; reproduced with permission from (Liu et al., 2021). **(B)** Schematic diagram of the preparation process of the triple-enhanced SERS MXene-AuNCs-FOTS platform; reproduced with permission from (Liu et al., 2023e). **(C)** Schematic diagram of MXene-coated Ag nanoparticles, and AFM images of Au particle hybrids coated with MXene in the SERS platform; reproduced with permission from (Yoo et al., 2022).

MXenes are capable of interacting with noble metal nanoparticles via electrostatic interactions for large-scale loading of nanoparticles, which facilitates combining the benefits of noble metal nanoparticles and MXene on SERS substrates. In general, noble metals, such as Au, Ag, and rarely Pd, have been coated on MXene by different methods to facilitate SERS detection of target analytes due to their stability in air. The two widely accepted SERS enhancement mechanisms are electromagnetic enhancement and chemical enhancement (Sharma et al., 2012). Satheeshkumar et al. found that the noble metal/MXenes combined substrate had high sensitivity and the enhancement factor could reach 10^4^–10^5^, which was the consequence of the synergistic effect of the electromagnetic enhancement of the noble metal and the chemical enhancement of MXenes (Satheeshkumar et al., 2016). It is worth noting that MXene and noble metals are the result of synergistic enhancement of the two, respectively. In the case of 2D SERS materials, for example, the electromagnetic enhancement mechanism has the main contribution to the total SERS enhancement, showing 10^4^–10^11^ enhancement, whereas the chemical enhancement factor is only 10^1^–10^7^, but it can transfer charge states between nanoparticles and molecules (Maitani et al., 2009). Thus, the hybridization of MXene with noble metal nanoparticles contributes to both mechanisms. A study investigated the enhancement effect of Ti_3_C_2_T_X_-Ag composites with different Ag contents as SERS substrates. The results showed that the spherical Ag nanoparticles on the surface and interlayer of Ti_3_C_2_T_X_ nanosheets became more tightly aggregated with the increase in Ag nanoparticle content. The highest value of the SERS enhancement factor appeared on the sample with the largest specific surface area (Xue et al., 2023). This study demonstrated that the tight stacking of Ag nanoparticles led to a significant enhancement of the surface plasmon resonance coupling, further realizing a substantial enhancement of the SERS signal. As shown in Figure 2A, the electrostatic self-assembly method could be used to prepare the Ti_3_C_2_T_X_-Ag nanoparticle hybrid biosensor, and the citrate-coated Ag nanoparticles were deposited upon the negative surface of the Ti_3_C_2_T_X_ sheet by diallyl dimethyl ammonium chloride (PDDA) polymer. This platform demonstrated acceptable homogeneity, long-term stability, and outstanding SERS performance (Liu et al., 2021).

As a representative plasma nanoparticle, Au is a unique plasma nanoparticle with highly curved surface characteristics, tunable plasma absorption bands, and remarkable chemical stability, which make it an effective SERS substrate. Au nanoparticles can be easily assembled on the surface of MXene by electrostatic forces, and the highly sensitive hotspot constructed by Au nanoparticles is utilized to enhance the electromagnetic mechanism, finally achieving ultra-high SERS performance in complex environments (Xie et al., 2019; Liu et al., 2023d). The MXene-Au nanocubes sensor demonstrated robust quantification and susceptible SERS detection of low analyte concentrations, thanks to the super-hydrophobic treatment of Au nanocubes-loaded MXene to eliminate the “coffee ring” effect and to effectively enrich the target analytes during the drying process. The preparation process of MXene/AuNCs-FOTS (1H,1H,2H,2H-perfluoro-octyltriethoxysilane) membranes was shown in Figure 2B, and the substrate synthesized by the seed growth method helped to generate highly sensitive SERS signals (Liu et al., 2023e). Unlike the conventional method of exposing Au nanoparticles on the MXene surface, conformally coating a single layer of MXene coating on a large-area colloidal Au nanoparticle 2D assembly also allowed for designing a unique SERS substrate. The highly conductive monolayer of MXene encouraged the charge transfer properties between the SERS substrate and the analyte, and the deposited MXene layer had a non-zero extinction coefficient in the spectrum, which helped to form a strong plasma in the vicinity of the Au nanoparticles, ultimately realizing a SERS enhancement factor of up to 1.6 × 10^10^. These MXene-coated Au-particle hybrids were prepared using the method illustrated in Figure 2C (Yoo et al., 2022).

Co-coating of multiple noble metals on MXene substrates is also widely used for SERS detection. One-step hybridization was processed for the synthesis of high-sensitivity Ti_3_C_2_T_X_ composites packed with multiple noble metals (Au, Ag, and Pd) (Satheeshkumar et al., 2016). Ti_2_C-Au-Ag nano shuttlesubstrates (NSs) prepared with ultrasonic dispersion showed large and rough surfaces, superb electrical conductivity, remarkable electrochemistry reactions, substantial Raman enhancement, and excellent stability. Notably, this experiment compared the SERS performance of Ti_2_C-Au-Ag nanohybrids with that of single Ti_2_C and Au-Ag NSs substrates, and the outcomes demonstrated that the SERS signals of Ti_2_C-Au-Ag NSs substrates significantly increased and were much more potent than those of the other two substrates. The potential explanation for the enhanced effect is the electromagnetic enhancement generated by localized electromagnetic fields produced by Au-Ag NSs as a hot spot (Zhu et al., 2021). Hongyan Bai’s team found that Ti_3_C_2_T_X_-Pd nanosolutes prepared using a two-step method can strongly catalyze the reduction of HAuCl_4_ by H_2_O_2_ to generate Au nanoparticles, and the synthesized Ti_3_C_2_T_X_-Pd-Au substrate exhibited a strong SERS effect, resonance Rayleigh scattering (RRS), and surface plasmon resonance absorption effect (Abs) (Bai et al., 2022). These studies broaden the design ideas of high-performance MXene substrates and provide more possibilities for developing sensitive SERS detection platforms.

## 3 Practical applications of MXene in SERS

Since the groundbreaking discovery of graphene in 2004, there has been a growing surge of interest in 2D materials owing to their remarkable chemical and physical properties (Novoselov et al., 2004). MXene has many potential applications among 2D materials due to its excellent SERS activity, biocompatibility, and environmental friendliness (Chen et al., 2020). Owing to the outstanding advantages of SERS, such as high sensitivity, high stability, and narrow spectral bandwidth, SERS detection based on MXene could be used in many scenes in production and life (Martinez Pancorbo et al., 2021; Tyagi et al., 2023).

### 3.1 Biomedical sensing

**FIGURE 3:**
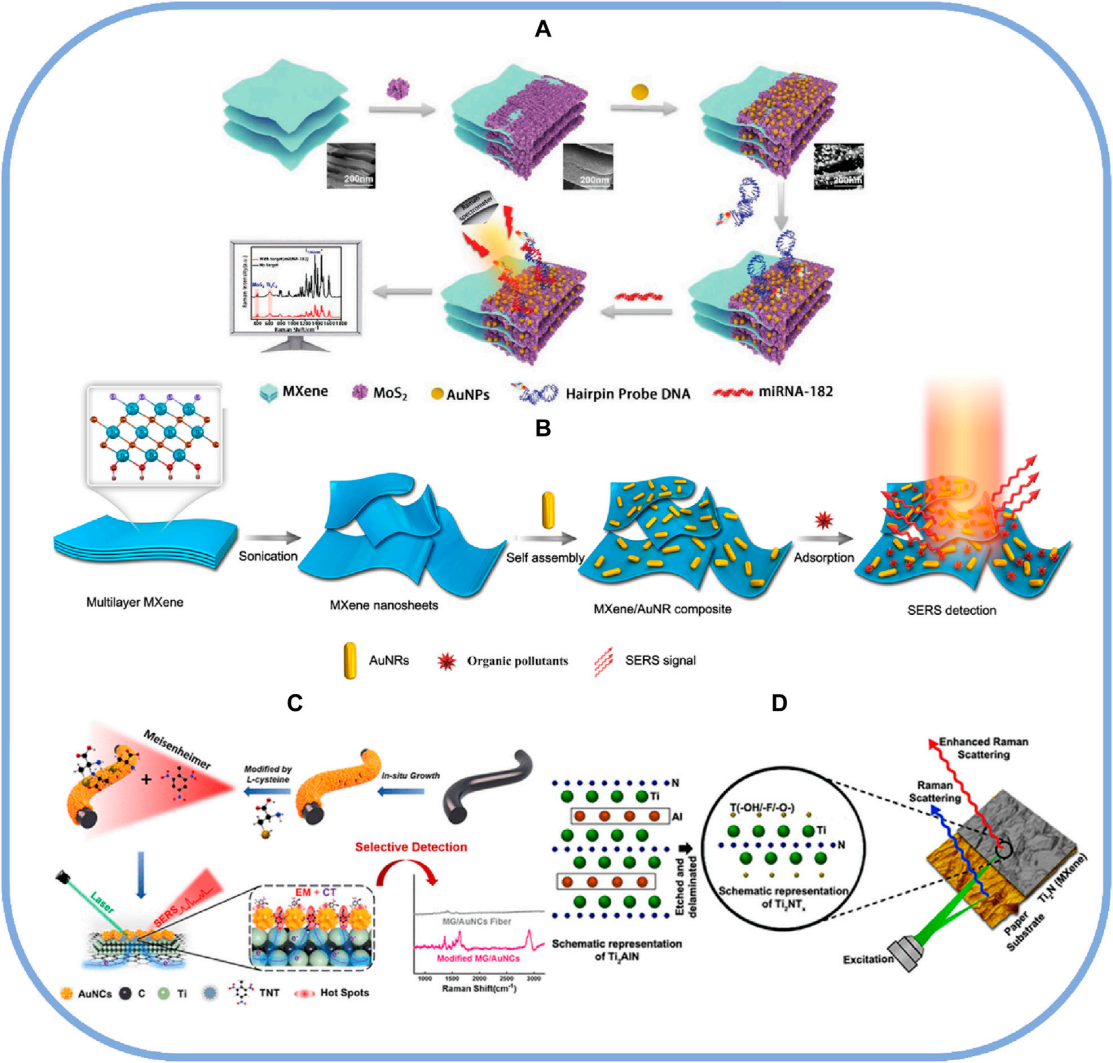
**(A)** Schematic diagram of the SERS platform based on MXene-MoS_2_-Au was prepared using collaborative calibration for ultra-sensitive detection of miRNA-182; reproduced with permission from (Liu et al., 2020a). **(B)** Schematic of the preparation strategy and application of Ti_3_C_2_T_X_-Au composites as SERS substrates; reproduced with permission from (Xie et al., 2019). **(C)** Schematic diagram of the wet spinning process for the preparation of Ti_3_C_2_T_X_-graphene-Ag nanoparticle fiber-optic sensors; reproduced with permission from (Liu et al., 2023d). **(D)** Schematic of the preparation of a SERS substrate based on Ti_2_NTx by loading it on paper to realize the detection of trace explosives; reproduced with permission from (Soundiraraju and George, 2017a).

Cancer is the second-leading cause of death after cardiovascular disease and one of the biggest health problems today. Therefore, early diagnosis of cancer becomes crucial. The primary method in the process of early diagnosis is the detection of malignant tumors through cancer biomarkers. The differentiation and progression of benign and malignant tumors are related to the concentration of carcinoembryonic antigens in plasma. A novel sandwich-type immunosensor with 4-mercaptobenzoic acid-labeled MoS_2_-Au nanoparticles as a CEASERS tag and hierarchical Ti_3_C_2_T_X_ functionalized with Fe_3_O_4_-Au nanoparticles as a magnetic support substrate for SERS had demonstrated to be useful for carcinoembryonic antigen (CEA) assays, providing an essential alternative biosensor for clinical diagnosis (Medetalibeyoglu et al., 2020). Another biomarker strongly associated with cancer is miRNA-182. As shown in Figure 3A, a co-calibrated SERS approach using ternary systems and MXene-MoS_2_-Au nanoparticles could achieve ultra-sensitive detection of miRNA-182, creating a linear detection window of miRNA-182 from 10 a.m. to 1 nm with an ultra-low detection limit of 6.61 a.m. Vertical MoS_2_ nanosheets anchored to layered MXene provided uniformly ordered sites for housing Au nanoparticles as hot spots and for adsorbing hairpin probe DNA via Au-S bonds. This strategy also had ultra-high linear goodness of fit, excellent selectivity, and high reproducibility (Liu et al., 2020a). In addition, microfluidics has been proposed in combination with SERS technology, and this was because the use of TiO_2_-Nb_2_C as a SERS substrate in combination with a microfluidic chip allowed the construction of an invasion model for real-time monitoring of glioma invasion, which could enable the targeted monitoring of the glioma biomarker vascular endothelial growth factor. In the meantime, repeated transfers of excitation electrons caused TiO_2_-Nb_2_C to exhibit considerable Raman signal augmentation under the excitation of a laser with a wavelength of 785 nm (Zhao et al., 2023). However, monitoring malignant tumors alone is not enough, and anticancer drug delivery technologies have become another hot topic of discussion. Combining the unique advantages of MXene and the excellent SERS properties of noble metal materials, the anticancer drug doxorubicin (DOX) was loaded onto the surface via 4-MBA using Ti_3_C_2_T_X_-Ag nanoparticle membranes as carriers. The DOX-loaded nanoparticle membrane was triggered by glutathione (GSH), and the addition of GSH triggered a thiol-exchange reaction, which led to the detachment of 4-MBA from the membrane surface and facilitated the efficient release of DOX. The final tracking and monitoring were realized by SERS (Chen et al., 2023). This study provides the possibility of utilizing membranes with three-dimensional structures as scaffolds for loading and releasing drugs in biotherapeutics.

Faced with the worldwide health problem of bacterial infection, we must develop a multifunctional detection platform that can detect bacteria rapidly and sensitively and inhibit or kill them effectively. Au nanoparticles were assembled on the surface of Ti_3_C_2_T_X_ by electrostatic interaction, and the synthesized Ti_3_C_2_T_X_-Au nanocomposites were helpful in rapid bacterial detection and photothermal sterilization. It is worth noting that after being exposed to 808 nm light for just 6 min, Ti_3_C_2_T_X_-Au nanoparticles had strong antibacterial activity against both Gram-positive *Bacillus subtilis* and Gram-negative *Escherichia coli*, with bactericidal ratings of 99.25% and 100%, respectively. In addition, this multifunctional nanocomposite material could rapidly detect bacteria and be used for antibacterial and photothermal sterilization (Yu et al., 2021). It is promising to tackle bacterial infections, especially those with antibiotic-resistant strains. Photoelectrochemical and SERS dual-mode biosensors have been suggested as a sensitive and precise method of detecting low concentrations of *Staphylococcus aureus* in the early stages of infection, which coupled with carbon nitride nanosheets C_3_N_4_-Au nanoparticles, sustained recognition of gas pedals combined with induced DNA walker coupling for the bacterial infection (Liu et al., 2023b). MXene also has broad applications in other biomedical fields. For example, the glucose concentration in diabetic patients' tears could be measured using a flexible SERS substrate created by growing Au nanoparticles on the surface of Ti_3_C_2_T_X_ nanosheets using a self-assembly technique, which had a minimum detection concentration of 0.39 μM, overcoming the limitations of the traditional blood glucose detection and creating a sensitive and noninvasive detection for SERS substrates based on MXene. The chemical enhancement brought about by the charge transfer between the MXene and dye molecules and the electromagnetic enhancement supplied by the Au nanoparticles were responsible for the SERS enhancement (Cui et al., 2022). In addition, TiC-Au nanoparticle-based SERS substrates prepared by a photoreduction process were used to detect the antipsychotic drug chlorpromazine in human biological fluids. Hot spots were created in the particle gap of Au nanoparticles bonded to the TiC surface by varying the photoreduction duration, and these hot spots significantly increased the amplification of the Raman signal. (Barveen et al., 2022).

### 3.2 Food safety inspection

Someone often overuses pesticides in production and life, causing harm to human health through biological enrichment. Conventional tools for detecting pesticides in food were less effective due to their low flexibility and stability. In recent years, we have recognized SERS technology as a powerful tool to address these issues, providing fast measurement times, smaller instrument sizes, analyte fingerprinting information, and non-destructive sampling. SERS substrates based on MXene can detect various contaminants in food, such as pesticides, antibiotics, and food additives. As shown in Figure 3B, for the purpose of detecting organic pollutants like fumonisins, Ti_3_C_2_T_X_-Au composites prepared through strong electrostatic interactions formed uniform SERS hotspots on the surface of Ti_3_C_2_T_X_ with a LOD of 10^−10^ M, which was significantly lower than that of the U.S. Environmental Protection Agency (Xie et al., 2019). The SERS substrate of natural β-cyclodextrin-coated silver nanoparticles modified with Ti_3_C_2_T_X_ nanoparticles developed by the one-pot method was used for the selective determination of the food additive erythrosin B. The linear detection range was 1–100 mg/L, and the LOD was 30 μg/L. It could rapidly analyze erythrosin B in dyed food samples within 15 min, and the recovery rate was as high as 78.1%–113.2%. This was made possible by the presence of β-cyclodextrin under alkaline conditions, which resulted in a dense and uniform distribution of Ag nanoparticles on the Ti_3_C_2_Tx nanosheets (Lai et al., 2021). Robust substrates such as these are valuable for effective and rapid SERS analysis of food safety issues.

Flexible platforms are more appropriate for real-world inspection applications since they allow for effective sampling and quick analysis in the field as compared to rigid platforms. *One work used* polydimethylsiloxane (PDMS) flexible substrate could detect 4-MBA at a LOD of 10^−11^ M. Ti_3_C_2_T_X_’s capacity to prevent Ag nanoparticle oxidation might help to localize the molecules under test within the hotspot. Crucially, the substrate’s flexibility made it possible to directly collect samples from uneven surfaces, like fruits and vegetables, in order to identify traces of pesticide residues. The combination of thiabendazole and 4-MBA was also detectable by the substrate (Xiong et al., 2022). Another Ti_3_C_2_T_X_-Ag flexible substrate extracted and detected thiabendazole, fumonisin, methyl parathion, and their mixtures using a transparent and versatile PDMS-based platform. A considerable number of hot spots were added to the hybridized film by the large Au nanoparticles produced by the self-reducing process and electrocoupling replacement, which significantly improved the SERS detection. Notably, the lowest concentration of 2.8 ng/mL detected by rubbing on the surface of a tomato was less than the maximum amount of fresh fruits and vegetables that the EU allows (Xiong et al., 2023). The creation of adaptable systems for quick multiplexed analysis of target molecules in real samples is made possible by these new insights.

Aptamer and optical fiber sensors based on SERS have recently attracted widespread attention. One work used a SERS aptamer sensor developed by Au-Ag Janus nanoparticles and MXene to quantitatively detect ochratoxin A in wine samples (Zheng et al., 2019). As shown in Figure 3C, Ti_3_C_2_T_X_-graphene-Ag nanoparticle fiber-optic sensors with outstanding SERS performance may be built for fast multiplexed sensing of pesticide residues in fruit peels, with a detection error of less than 7.3%, by combining wet spinning and self-assembly at the oil-water interface. The use of graphene fiber offered substantial adaptability and charge transfer enhancement for the SERS platform and allowed Au nanoparticles to grow *in situ* on the surface and form high sensitivity hot spots, thereby improving the SERS performance and durability of the substrate in complex environments. They could also effectively recognize nikethamide (a prohibited stimulant), crystal violet (a fish drug), and methylene blue (an organic pollutant) compounds (Liu et al., 2023c).

In addition to having the benefits of a single mode, the dual-modal analysis approach can also mitigate its drawbacks. A dual-mode probe combining SERS technology and electrochemistry could achieve rapid, qualitative, and sensitive contaminant detection by taking advantage of each. *Vibrio* traumaticus in seafood could be detected with extreme sensitivity thanks to the dual mode electrochemiluminescence and SERS combined immunoassay technique based on Ti_3_C_2_T_X_. The Faraday cage sensor was designed in such a way that all electrochemiluminescence signal tags were electrochemically activated (Wei et al., 2021). In a recent investigation, Ag-Cu_2_O nanoparticles with electroactive and SERS activity were assembled with MXene nanosheets to form an aptasensor with parallel surfaces, good electrical conductivity, and intrinsic Raman properties to detect tetrodotoxin. The aptasensor has both electrochemical and signal programming modes. For electrochemical experiments, 2D MXene offers a wide surface area for aptamer binding and high conductivity to speed up electron transmission (Yao et al., 2023). The thermal design of plasma metal-semiconductor inhomogeneous nanocomposites shows significant promise in building multimodal biosensing platforms for quantitative and accurate detection of analytes in complex systems.

### 3.3 Environmental detection

Environmental pollution is a global issue, and the necessity to develop practical and sensitive analytical techniques for identifying different environmental contaminants has become important due to the rising concern about organic pollutants in soils and rivers. The application of MXene materials for the susceptible, rapid, and accurate detection of hazardous chemicals in the environment has promising applications. Polychlorinated biphenyls are a category of semi-volatile, difficult-to-degrade, synthetic persistent organic pollutants of chlorinated aromatic hydrocarbons that may spread over great distances. Ag-Ti_3_C_2_T_X_ substrates were prepared by modifying ultrathin MXene nanosheets on the outermost layer of Ag nanorods, which could be utilized for the detection of single-component and multi-component polychlorinated biphenyls (PCBs) in actual soil samples with high recoveries (single-component recoveries of 90.3%–91.6%, multi-component recoveries of 108.1%–106.5%). Because the MXene substrates inhibited the oxidation of silver nanorods, they offered high stability and repeatability (Yang et al., 2018). Yuting Ye’s team synthesized highly crystalline monolayer Ti_3_C_2_ nanosheets using improved chemical stripping and microwave heating methods, achieving a LOD of 10^−11^ M combined with common environmental contaminants including bisphenol A, trichlorophenol, and azo dyes, which made the MXene substrate far superior to the detection of the majority of semiconductor substrates and was even comparable to precious metal substrates. At the same time, this was the first time that approximate single-molecule imaging has been realized on a non-precious metal SERS substrate (Ye et al., 2020). Another Ti_3_C_2_-functionalized superlattice SERS substrate allowed the detection of many fish drug residues in pond water. This test was very sensitive and consistently repeatable, and it increased substrate adsorption of a variety of fish medicines when Ti_3_C_2_ was deposited on phases of vertically self-assembled bimetallic nanocuboids (Miao et al., 2021). As an essential raw material, some have widely used phenolic compounds in chemical production, among which catechol has high toxicity and belongs to class 2B carcinogens. In a recent study, layered Ti_3_C_2_ films were prepared by electrodeposition on aluminum plates, and Ag nanoparticles were altered using *in situ* reduction on Ti_3_C_2_ sheets. These Ti_3_C_2_ membranes exhibited a strong SERS effect, excellent signal repeatability, and high reproducibility for trace catechol in water. More notably, the substrate had outstanding salt tolerance and was extremely perceptive to catechol traces in saltwater, with a LOD as high as 10^−7^ mol/L (Liu et al., 2023b). These findings suggest that the MXene membrane has great promise for the timely detection of catechols in saltwater and that it will eventually be produced and used on a large scale.

Heavy metal ions and partial cations harm ecology and human health because they are non-degradable and tend to accumulate in the environment or in living organisms. A field-effect transistor sensor using Ti_3_C_2_T_X_ as a channel material could detect Hg^2+^ in water, which showed a fast selective response to Hg^2+^. In addition, the sensor achieved surprising Hg^2+^ detection performance in high salinity environments, which was conducive to its application in fundamental water analysis. It had essential application prospects for on-site monitoring and risk assessment of Hg^2+^ in water systems (Hao et al., 2021). Another Mo_2_CT_X_-Fe_2_O_3_-Ag hybrid nanostructure could detect arsenic (As), a harmful cationic molecule in wastewater. The particular attachment of the positively charged ion methylene blue to the negatively charged MXene surface allowed for the chemical enhancement (Sakir et al., 2023).

2, 4, 6-Trinitrotoluene (TNT) deposited in the environment is toxic to animals and humans and can cause skin damage, liver abnormalities, and anemia. Low sensitivity, high instrumental costs, and long determination processes characterize traditional TNT detection methods. As shown in Figure 3D, Bhuvaneswari Soundiraraju’s team loaded Ti_2_NT_X_ onto paper, silicon, and glass to prepare different SERS substrates based on them. The results showed that Ti_2_NT_X_ had the highest enhancement intensity of 10^12^ on the paper-based substrate, which was attributed to the fact that the paper substrate could effectively concentrate the analyte, resulting in a stronger Raman signal. This SERS substrate could be used for the detection of trace explosives (Soundiraraju and George, 2017a). Recently, a tri-modal peptide detection platform was made available for the ultrasensitive detection of TNT. This method was used *in situ* high-frequency etching to prepare Ti_3_C_2_ nanosheets and stabilized and powerfully catalyzed CO reduction of PdCl_2_-prepared Ti_3_C_2_-Pd nanosolvents. The researchers constructed a sensitive, simple, and inexpensive three-mode peptide detection platform based on nanocatalytic and peptide reactions to detect TNT in wastewater and soil samples, with relative standard deviations (RSDs) ranging from 6.22% to 8.77% and recoveries spanning from 98.7% to 106%. In addition, the biosensing platform could also be used to detect glyphosate and estradiol, respectively (Bai et al., 2022). In conclusion, the SERS strategy mentioned above based on MXene substrate provides many practical solutions in various fields of production and life and has broad application prospects.

## 4 Conclusion and outlook

MXene is a newly developed biosensing nanomaterial for SERS substrates that has advanced at an unparalleled rate in the last few years. In this review, we summarize the strategies of MXene used as a SERS substrate alone and in combination with noble metal nanoparticles and conclude the applications of SERS sensors based on MXene substrates in various fields, including biomedical sensing, food safety detection, and environmental monitoring. Various application scenarios have proved that MXene, as a SERS substrate, can generate an ultra-high enhancement factor for rapid and sensitive trace detection, which has broad application potential. However, there are currently greater challenges in extending the life cycle and reducing the toxic effects of MXene materials. Surface modification of MXene with various nanostructures in the future may improve biocompatibility, stability, and recyclability and reduce the cytotoxicity of samples. Another concern is improving the adhesion and selectivity between the noble metal nanoparticles and the substrate material, an issue that may limit the broad application of SERS technology. Adding reducing agents and stabilizers to the preparation process is expected to solve the problem of poor adhesion, but we should further explore more efficient strategies. We hope this article on the current status of SERS applications based on MXene materials will generate more interest in this emerging field. With the development of nanotechnology, the 2D material MXene will be used in a broader range of detection scenarios, bringing better development to our lives.
